# Small rodent monitoring at Birkebeiner Road, Norway

**DOI:** 10.3897/BDJ.11.e105914

**Published:** 2023-09-11

**Authors:** Magne Neby, Harry Andreassen, Cyril Pierre Milleret, Simen Pedersen, Ana-Maria Peris Tamayo, David Carriondo Sánchez, Erik Versluijs, Barbara Zimmermann

**Affiliations:** 1 Faculty of Applied Ecology, Agricultural Sciences and Biotechnology, Campus Evenstad, Inland Norway University of Applied Sciences, Koppang, Norway Faculty of Applied Ecology, Agricultural Sciences and Biotechnology, Campus Evenstad, Inland Norway University of Applied Sciences Koppang Norway; 2 Faculty of Environmental Sciences and Natural Resource Management, Norwegian University of Life Sciences, Ås, Norway Faculty of Environmental Sciences and Natural Resource Management, Norwegian University of Life Sciences Ås Norway; 3 Faculty of Biosciences and Aquaculture, Nord University, N-8049 Bodø, Norway Faculty of Biosciences and Aquaculture, Nord University N-8049 Bodø Norway

**Keywords:** arvicolinae, vole, shrews, trapping, occurrence, database, dissection

## Abstract

**Background:**

Northern small mammal populations are renowned for their multi-annual population cycles. Population cycles are multi-faceted and have extensive impacts on the rest of the ecosystem. In 2011, we started a student-based research activity to monitor the variation of small rodent density along an elevation gradient following the Birkebeiner Road, in southeast Norway. Fieldwork was conducted by staff and students at the University campus Evenstad, Inland Norway University of Applied Sciences, which has a long history of researching cyclic population dynamics. The faculty has a strong focus on engaging students in all parts of the research activities, including data collection. Small rodents were monitored using a set of snap trap stations. Trapped animals were measured (e.g. body mass, body length, sex) and dissected to assess their reproductive status. We also characterised the vegetation at trapping sites.

**New information:**

We provide a dataset of small rodent observations that show fluctuating population dynamics across an elevation gradient (300 m to 1,100 m a.s.l) and in contrasting habitats. This dataset encompasses three peaks of the typical 3-4-year vole population cycles; the number of small rodents and shrews captured show synchrony and peaked in years 2014, 2017 and 2021. The bank vole *Myodesglareolus* was by far (87%) the most common species trapped, but also other species were observed (including shrews). We provide digital data collection forms and highlight the importance of long-term data collection.

## Introduction

Voles and lemmings in boreal, alpine and arctic ecosystems are renowned for their multi-annual population cycles (Elton 1924, Hansson and Henttonen 1985, Kendall et al. 1999). The fluctuating population dynamics amplify the population’s integral roles in the ecosystem food web (Boonstra et al. 2016) as vectors of diseases, prey and plant consumers Nystuen et al. 2014, Magnusson et al. 2015, Kouba et al. 2021. Many decades of studies on population cycles have brought insights into the complexity of mechanisms involved in the dynamics and ecology of populations (Stenseth 1999, Myers 2018, Oli 2019). However, many questions remain to understand the generality of small rodent population dynamics (Andreassen et al. 2021).

The focus of this study is the understanding and exploration of the population dynamics of arvicoline rodent species, particularly the field vole *Microtusagrestis* (Linnaeus, 1761) and the bank vole *Myodesglareolus* (Schreber, 1780), which are amongst the most widespread and abundant mammals in the European boreal biome. Moreover, the ongoing changes in climate and biodiversity, particularly warmer winters, are expected to affect these population dynamics and, consequently, the role these small rodents play in the ecosystem (Cornulier et al. 2013). In general, long-term studies are much needed to understand such effects; especially when controlling for phase dependence in multi-annual cycles. Here, we contribute to the research on population dynamics and the intricate mechanisms involved, by providing a dataset that encompasses three population cycle peaks. We include data on small mammal occurrences, physiological measures on captures and habitat descriptions.

## General description

### Purpose

Assessing changes can only be observed by comparing the new state with a previous one. Thus, systematic long-term data collection efforts are vital to reveal changes in nature or the lack thereof. Nevertheless, due to long-term data’s innate high degree of replication, the credibility and usefulness of such time series are high in research, management and policy (Clutton-Brock and Sheldon 2010, Magurran et al. 2010). Ironically, as the need for long-term time series increases, the persistence of established long-term studies is weakened and the establishment of new ones is rare (Hughes et al. 2017).

Evenstad is located in the southeast of Norway and is a campus at Inland Norway University of Applied Sciences. Evenstad has a long history in ecological investigations (e.g. Mathisen et al. (2012), Neby et al. (2021)), including researching population dynamics (e.g. Pedersen et al. (2011), Johnsen et al. (2017)). Furthermore, the faculty has a strong focus on engaging students in all parts of the research activities, including research data collection. In 2011, we started a student-based research activity to monitor small rodent populations. After more than ten years, the time series is still young in terms of observing cyclic phenomena. Nonetheless, the data include three high-density periods (i.e. peaks). We hope this paper will motivate long-term maintenance of the time series and facilitate data and knowledge sharing.

## Project description

### Title

Birkebeinervegen monitoring (alias Birkebeinervegen rodent trapping).

### Personnel

**Personnel**: Over different years, the co-authors have led the sampling in the field and/or lab, with a yearly turnover of student participation.

**Figure 1.**
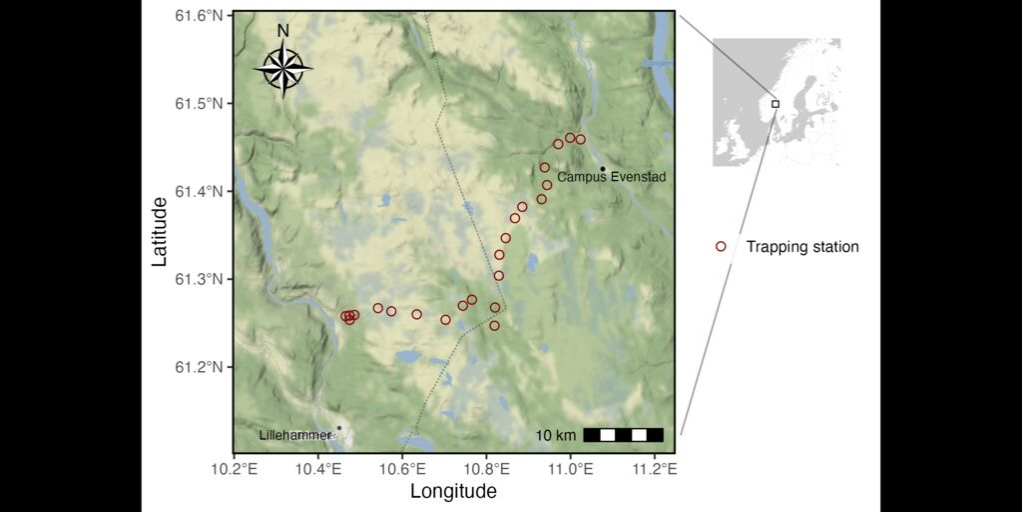
Study area and the 23 trapping station locations (red circles) situated along the Birkebeiner Road. Each station contains a transect of 10 traps with fixed locations. The vegetation descriptions were taken close to the traps. The captures were further described and dissected at the University campus Evenstad (marked with a black dot).

**Study area description**: The study area is located in the southeast of Norway, in Innlandet Municipality (61°N, 11°E, Fig. 1). Here, arvicoline small rodents are known to exhibit cyclic population dynamics (Andreassen et al. 2020, Sonerud 2022) and the area is characterised by a semi-humid and continental climate. The study area is situated across an elevation gradient following the east-west orientated Birkebeiner Road with gradients in temperature, precipitation and vegetation (Table 1). The area is dominated by mixed coniferous forests with Norway spruce *Piceaalbies* and Scots pine *Pinussylvestris* at low altitudes and more open areas with mountain birch *Betulapubescens* at higher elevations. The understoreys are dominated by dwarf shrubs, such as the bilberry *Vacciniummyrtillus* in the low elevations and lichens and grasses higher up.

**Funding**: This research was part of the BEcoDyn project supported by Hedmark University of Applied Sciences and a grant from the Norwegian Research Council (NFR project 221056) to H.P.A. Remaining funding from INN—Inland Norway University of Applied Sciences.

## Sampling methods

### Study extent

**Study design**: The Birkebeiner Road connects the two large valleys Gudbrandsdalen and Østerdalen from east to west over approximately 60 km (40 km straight line distance). The locations for the trapping stations were selected to obtain 100 m a.s.l. intervals between each station ranging from 300 m to 1,100 m a.s.l. (Fig. 1). This resulted in stations being between 0.5 and 4 km apart. This minimised the chances of trapping stations overlapping home ranges of small rodents.

In the initial setup for each trapping station, starting 10 m from the road, 10 metal snap traps (numbered 1-10, with trap 1 being closest to the road) were placed systematically 10 m apart along a transect at a right angle from the road. These trap locations were registered using handheld GPS devices (ca. 5 m accuracy), marked with coloured ribbons that were left out all year and their coordinates were reused the following years. The traps were placed in the understorey at the exact GPS location, however, slightly adjusted from 2022, when the traps were placed in the understorey in the most suitable location (i.e. close to holes, rock boulders, tree roots etc.) within one metre from the GPS location in order to maximise catches within the microsite.

**Small mammal data**: The trapping sessions consisted of five days of fieldwork. The trapping session was started by activating and baiting the traps with pieces of carrots mixed with peanut butter during the first morning. The three following mornings, all traps were checked and, if necessary, re-baited and/or re-activated. During these procedures, the trap’s status was noted (e.g. animal captured, broken trap etc., see a complete set of variables and definitions in the Data Resources section below in the DynamicProperties description in the Event dataset) and trapped animals were collected. On the last day of the session, traps were monitored as usual and retrieved from the field.

**Figure 2.**
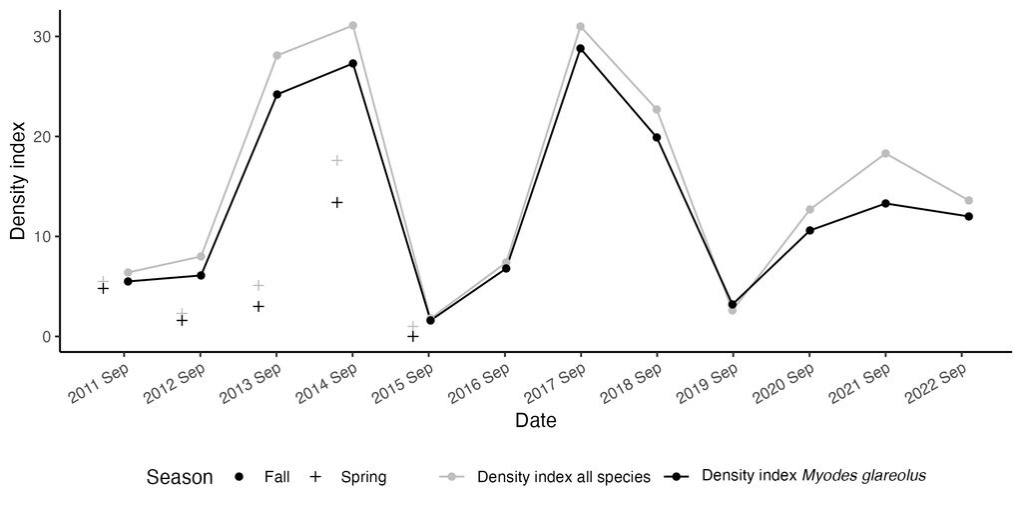
The density index is estimated by the number of captured animals (grey) and captured bank voles *Myodesglareolus* (black) per 100 trap nights for annual autumn trappings (filled circle). The first years of trapping also included trapping during the spring season (+).

We carried out trapping sessions in autumn from years 2011 onwards and also in spring during the years 2011–2015. This translates into a total effort of 690 trap-nights per trapping session (see details in Table 2) with large inter-annual variation in the number of caught animals (Fig. 2, Table 2) and with the bank vole (*Myodesglareolus*) as the most common catch (87%).

The collected animals were either brought into a laboratory immediately or frozen at -20^o^C until dissection. Here, the animals were examined further including dissection for reproductive trait measures (see a complete set of variables and their description in Table 3). By using the variables eventID, occurrenceID or locationID, the datasets can be joined.

**Vegetation data**: Within a five-metre radius of each trap, we monitored the vegetation by describing the dominant habitat, tree layer, bush layer and field layer. We characterised these variables according to the descriptors given in Table 4. In 2020, each station’s habitat was described in further detail (Table 5), named with the prefix "ext" in the Extendedmeasurementorfact dataset.

**Data availability**: The data are available on Dataverse (DOI: https://doi.org/10.18710/OOJYQ0) and consist of three datasets that can be joined using the variables eventID, occurrenceID or locationID. The data and R script to ease download and import (including producing Fig. 2) are available at https://gitlab.com/becodyn/birkebeiner. Updates from future monitoring will be available on these services.

**Quality control**: We used standardised field forms to note observations which were followed by import to MS Excel (2011-2019 and 2021) and with predefined digital forms using KoboCollect (https://www.kobotoolbox.org/) from 2020 and onwards (with the exception of 2021) to reduce transcribing errors. The digital forms are available as .XML in the repositories and can be imported to KoboToolbox, ODK or similar services. Permits for trapping are given by The Norwegian Ministry of Climate and Environment.

**Sources of error**: Snap trapping provides only a relative density index. There are also local sources of error that potentially affect within and between-year values, such as: 1) trapping in various weather conditions affecting trapping success, 2) trap placement in more/less risky/suitable microhabitats, 3) trap placement in general could be an issue in spatio-temporal analysis, 4) molar teeth were not always checked on *Microtus* sp., thus there may be species level uncertainty between species identified as *M.oeconomus* and *M.agrestis*.

## Geographic coverage

### Description

**Description**: Birkebeiner Road, Innlandet County, Norway.

### Coordinates

61.460856 and 61.247073 Latitude; 11.023757 and 10.465131 Longitude.

## Taxonomic coverage

### Taxa included

**Table:** 

Rank	Scientific Name	Common Name
kingdom	Animalia	Animal
phylum	Chordata	
subphylum	Vertebrata	
class	Mammalia	
order	Rodentia	
order	Eulipotyphla	
family	Cricetidae	
family	Soricidae	
family	Muridae	
subfamily	Arvicolinae	
genus	*Apodemus*	
genus	*Lemmus*	
genus	*Microtus*	
genus	*Myodes*	
genus	*Myopus*	
genus	*Sorex*	
species	*Apodemusflavicollis*	Yellow-necked mouse
species	*Apodemussylvaticus*	Wood mouse
species	*Lemmuslemmus*	Norway lemming
species	*Microtusagrestis*	Field vole
species	*Microtusoeconomus*	Tundra vole
species	*Myodesglareolus*	Bank vole
species	*Myodesrufocanus*	Grey red-backed vole
species	*Myopusschisticolor*	Wood lemming

## Temporal coverage

### Notes

We monitored all trapping plots in the fall during the period from 07-06-2011 to 20-10-2022. During the years 2011-2015, we also performed a trapping session during the spring. The monitoring is planned to continue in the years ahead.

## Usage licence

### Usage licence

Creative Commons Public Domain Waiver (CC-Zero)

### IP rights notes

The dataset in the current work is licensed under a Creative Commons Attribution (CC-BY) 4.0 Licence.

## Data resources

### Data package title

Birkebeinervegen monitoring

### Resource link

DOI: https://doi.org/10.18710/OOJYQ0

### Alternative identifiers

https://gitlab.com/becodyn/birkebeiner

### Number of data sets

3

### Data set 1.

#### Data set name

Event

#### Data format

Darwin Core Archive

#### Character set

UTF-8

#### Download URL

https://doi.org/10.18710/OOJYQ0

#### Data format version

1.2

#### Description

A Darwin Core formatted file that describes an occurrence of an event, such as a trapping survey.

**Data set 1.** 

Column label	Column description
eventID	An identifier for the set of information associated with an Event. The values consist of the trapping station and the trap number separated by a T (Trap) and the date of the event. (Variable type: text)
eventDate	The date which an Event occurred in the format 'YYYY-MM-DD'. (Variable type: text).
locationID	An identifier for the set of Location information consisting of the trap station (1-23) and trap number (1-10) separated by T (Trap). (Variable type: text).
verbatimCoordinates	The verbatim original spatial coordinates of the Location. (Variable type: text).
verbatimCoordinateSystem	The spatial coordinate system for the verbatimCoordinates of the Location. (Variable type: text).
verbatimSRS	The spatial reference system (SRS) upon which coordinates given in verbatimCoordinates are based. (Variable type: text).
decimalLongitude	The geographic longitude (in decimal degrees). (Variable type: numeric).
decimalLatitude	The geographic latitude (in decimal degrees). (Variable type: numeric).
coordinateUncertaintyInMeters	The horizontal distance (in metres) from the given decimalLatitude and decimalLongitude describing the smallest circle containing the whole of the Location. (Variable type: numeric).
geodeticDatum	The ellipsoid, geodetic datum or spatial reference system (SRS) upon which the geographic coordinates given in decimalLatitude and decimalLongitude are based. (Variable type: text).
dynamicProperties	A list of additional measurements, facts, characteristics or assertions about the record. The keys (i.e. TrapReleased, BaitPresent, Capture, TrapRetrieved, TrapMoved, TrapConditionOK) and values (i.e. Yes or No) are separated by colons and properties separated by commas for a data interchange format, such as JSON. (Variable type: text).
minimumElevationInMeters	The lower limit of the range of elevation (altitude, usually above sea level), in metres. (Variable type: text).
maximumElevationInMeters	The upper limit of the range of elevation (altitude, usually above sea level), in metres. (Variable type: text).

### Data set 2.

#### Data set name

Occurrence

#### Data format

Darwin Core Archive

#### Character set

UTF-8

#### Download URL

https://doi.org/10.18710/OOJYQ0

#### Data format version

1.2

#### Description

A Darwin Core formatted file that describes the recorded instance of an organism at a particular time and place given in event.txt file. It includes information about the taxonomy and other relevant details. Further details on the trapped animals are given in the dataset Extendedmeasurementorfact and further described in Table 3.

**Data set 2.** 

Column label	Column description
eventID	An identifier for the set of information associated with an Event. (Variable type: numeric).
occurrenceID	An identifier for the Occurrence, here numbers in an ascending sequence from 1. (Variable type: text).
scientificName	The full scientific name, with authorship and date information, if known. Includes the name in lowest level taxonomic rank that can be determined. (Variable type: text).
taxonRank	The taxonomic rank of the most specific name in the scientificName. (Variable type: text).
sex	The sex of the biological individual(s) represented in the Occurrence. (Variable type: text).

### Data set 3.

#### Data set name

Extendedmeasurementorfact

#### Data format

Darwin Core Archive

#### Character set

UTF-8

#### Download URL

https://doi.org/10.18710/OOJYQ0

#### Data format version

1.2

#### Description

A Darwin Core formatted file that contains additional measurements or facts about the occurrences that are not included in the core occurrence.txt file, e.g. vegetation measurements included. The variable measurementType is further described in Tables 3, 4, 5.

**Data set 3.** 

Column label	Column description
measurementID	An unique identifier for the MeasurementOrFact here numbers in an ascending sequence from 1. (Variable type: text).
eventID	If relevant, an identifier for the set of information associated with an Event. Join with event.txt to include additional details, such as coordinates. (Variable type: text).
occurrenceID	If relevant, an identifier for the Occurrence. Join with occurrence.txt to include additional details, such as body mass of a trapped animal. (Variable type: text).
locationID	If relevant, an identifier for the location that can be joined with event.txt. (Variable type: text).
measurementDeterminedDate	The date on which the MeasurementOrFact was made. (Variable type: text).
measurementType	The nature of the measurement, fact, characteristic or assertion. The vocabulary uses three types of prefixes: 'anim' for animal measures; and 'min' for minimum and 'ext' for extended habitat measures, the latter two separating the two methods used for describing the habitat associated with locationID. These are further described in Tables 3, 4 and 5, respectively. (Variable type: text).
measurementValue	The value of the measurement, fact, characteristic or assertion. Values include text and numeric values depending on the MeasurementType, see Tables 4, 5. (Variable type: text).
measurementUnit	The units associated with the measurementValue, for example, body mass is given in grams (g). (Variable type: text).

## Figures and Tables

**Table 1.** Weather statistics in representative locations to the trapping transect area (MET Norway, 2022).

	**Western endpoint**	**High elevation point**	**Eastern endpoint**
Elevation (m a.s.l.)	500	1100	300
Weather station name	Lillehammer - Nordsetervegen	Sjusjøen - Storåsen	Evenstad
- Elevation (m a.s.l.)	562	930	255
- Temperature (^0^C)	4.2	2.1	4.0
- Mean daily precipitation (mm)	2.3	2.9	NA

**Table 2.** Trapping history with number of total captures during trapping season. When we were unable to find a trap or it was broken, these were subtracted from the default number of 690 trap nights (i.e. default 230 traps over three nights).

**Year**	**Month**	***Apodemusflavicollis***	***Apodemussylvaticus***	***Lemmuslemmus***	***Microtusagrestis***	***Microtusoeconomus***	***Microtus* sp.**	***Myodesglareolus***	***Myodesrufocanus***	***Myopusschisticolor***	***Sorex* sp.**	**Unidentified**	**Total captures**	**Number of trap nights**
**2011**	June	1	-	2	1	1	-	33	-	1	-	-	39	690
**2011**	September	-	-	-	-	-	-	38	6	-	2	-	46	689
**2012**	June	1	-	-	-	-	-	11	1	-	2	-	15	690
**2012**	September	1	1	-	-	-	-	42	2	-	9	-	55	690
**2013**	June	-	-	-	4	-	-	21	1	-	1	1	28	689
**2013**	September	-	1	-	12	-	-	166	-	-	3	3	185	686
**2014**	June	-	-	2	20	1	-	92	-	1	-	4	120	688
**2014**	September	-	-	1	13	2	-	186	-	-	8	-	210	682
**2015**	June	-	-	-	-	-	-	7	-	-	-	-	7	690
**2015**	September	-	-	-	-	-	-	11	-	-	1	-	12	685
**2016**	September	-	-	-	1	-	-	47	-	-	4	-	52	688
**2017**	September	-	-	1	9	-	-	184	-	-	2	1	197	639
**2018**	September	-	-	-	2	-	-	137	-	-	5	-	144	687
**2019**	September	-	-	-	-	-	-	22	-	-	-	-	22	685
**2020**	September	-	4	-	3	-	-	72	-	-	2	-	81	677
**2021**	September	-	2	-	23	-	-	92	-	-	4	3	124	690
**2022**	October	-	-	-	-	-	1	83	4	-	6	-	94	690

**Table 3.** The animals that were trapped were further analysed in the laboratory and several variables were measured. These measures are named with the prefix "anim" in Extendedmeasurementorfact dataset.

**Variable**	**Variable description**
MaturityOutside	An age/maturity approximation of the animal, either adult, juvenile or unidentified based on cues on the outside of the animal (Variable type: text)
BodyMass	The body mass of the animal is given in grams. (Variable type: numeric)
Tail	The length of the tail in mm (Variable type: numeric)
HeadWidth	The width of the head/skull in mm (Variable type: numeric)
BodyLength	The length of the whole body including the tail in mm (Variable type: numeric)
MaturityInside	Maturity approximation based on immature females having a transparent uterus and mature females having a milky-white uterus (Variable type: text)
LitterSize1	Placental scars were used to estimate litter size. First litter. Number of scars from the freshest litter (darkest scars) (Variable type: integer)
LitterSize2	Placental scars were used to estimate litter size. Second litter. Number of scars of the next litter back in time (Variable type: integer)
LitterSize3	Placental scars were used to estimate litter size. Third litter (Variable type: integer)
LitterSizeSummed	The total number of placental scars. Independent of litter (Variable type: integer)
EmbryoCount	Count of embryos, total (Variable type: integer)
EmbryoLength	Embryos were extracted from the body and measured in millimetres. Measured all and calculated average (Variable type: numeric)
EmbryoResorption	The number of embryo resorption. Small and less developed embryos are subject to resorption (Variable type: numeric)
TestesVisibleOutside	Visibly swollen testes on the outside prior to dissection (Present or absent) (Variable type: text)
TestesLength	Total length in mm of testes measured during dissection (Variable type: numeric)
Tubili_epididimysPresent	Tubili present in the epididymis or absent. The body next to the testes containing whitish coiled tube signifies maturity in males (Variable type: text)
Tubili_epididimys	Length of tubuli epididymis during dissection in mm (Variable type: numeric)
ObserverLab	An anonymised identifier of the observer in the lab (Variable type: integer)
ObserverField	An anonymised identifier of the observer during fieldwork (Variable type: integer)

**Table 4.** Vegetation measures performed during each trapping season. Here, the nearby surroundings of each trap were described using the following fixed variables. These measures are named with the prefix "min" in the Extendedmeasurementorfact dataset.

**Dominant habitat**	**Dominant tree layer**	**Dominant shrub layer**	**Dominant field layer**
Open	*Picea* sp.	*Picea* sp.	Bryophytes
Forest	*Pinus* sp.	*Pinus* sp.	Dwarf shrubs
Shrubs	Deciduous	Deciduous	Graminoids
-	*Juniperus* sp.	*Juniperus* sp.	Lichens
-	None	None	Herbs
-	-	-	Bare ground

**Table 5.** Extended vegetation measures were performed in 2020 and 2021. Here, the nearby surroundings of each trap were described in more detail using the following fixed variables. These measures are named with the prefix "ext" in the Extended measurement or fact dataset.

**Dominant habitat type**	**Forest cutting class**	**Cutting class description**	**Field layer cover variables**	**Field layer cover alternatives**
Forest	0	0: impediment (non-productive forest)	Bilberry	absent
Bog	1	1: fresh clearcut, ready for planting and regrowth	Cowberry	seldom < 5%
Shrub	2	2: young forest before first thinning, trees up to 10-12 m	Other heather	frequent < 5%
Alpine tundra	3	3: young forest in thinning stage	Grasses	5-25%
-	4	4: forest ready to be harvested	Herbs	25-50%
-	5	5: old-growth forest	Mosses	50-75%
-	-	-	Lichens	75-100%
-	-	-	Stones	-
-	-	-	Old wood	-
-	-	-	Bare ground	-
